# Virtual group consultations offer continuity of care globally during Covid‐19

**DOI:** 10.1002/lim2.17

**Published:** 2020-12-20

**Authors:** Fraser Birrell, Rob Lawson, Marianne Sumego, Jessica Lewis, Angela Harden, Tracey Taveira, John Stevens, Alison Manson, Linda Pepper, Jeannette Ickovics

**Affiliations:** ^1^ Medical Research Council Versus Arthritis Centre for Integrated Research into Musculoskeletal Ageing Newcastle University Newcastle upon Tyne UK; ^2^ Department of Rheumatology Northumbria Healthcare NHS Foundation Trust Ashington UK; ^3^ President, European Lifestyle Medicine Council Haddington UK; ^4^ Chairman, British Society of Lifestyle Medicine, East Linton UK; ^5^ Department of Internal Medicine & Geriatrics Cleveland Clinic Cleveland Ohio; ^6^ Department of Chronic Disease Epidemiology Yale School of Public Health New Haven Connecticut; ^7^ School of Health Sciences University of London London UK; ^8^ College of Pharmacy University of Rhode Island Kingston Rhode Island; ^9^ Department of Health and Human Sciences Southern Cross University Lismore New South Wales Australia; ^10^ Training Lead Group Consultations Ltd Glasgow UK; ^11^ Dean of Faculty Department of Rheumatology Northumbria Healthcare NHS Foundation Trust North Shields UK; ^12^ Yale‐NUS College Singapore; ^13^ Department of Social and Behavioral Sciences Yale School of Public Health New Haven Connecticut; ^14^ Department of Psychology Yale University New Haven Connecticut

**Keywords:** Empowerment, Group consultations, Shared medical appointment, Video group clinics, Virtual group consultations

## Abstract

Covid‐19 has led to virtual care (mainly telephone consultations) becoming a default worldwide, despite well‐documented shortcomings. Published evidence on virtual group consultations is limited, although interest and front‐line experience have grown substantially since pandemic onset. Unpublished data are summarised showing feasibility of transitioning care to this model across different countries, care settings and conditions. An international webinar series has supported development and sharing of best practice and representative data on spread and utilisation of virtual groups. This model of care creates time and space for more questions and answers, so once engaged patients become staunch advocates. Group care supports personalised care and lifestyle medicine, which is growing very rapidly. In the current context, even healthcare providers under pressure can implement virtual group consultations. Most virtual group consultations have a facilitator, so this allows roles to be extended and support education of both students and new team members. These can confer greater access, continuity of care, peer support and timely information about Covid‐19 and may result in better health outcomes. Given the rapid and widespread implementation of virtual care during this pandemic, data should be shared effectively and methodologically sound observational studies and clinical trials to test safety and effectiveness should be promoted now.

## 1 INTRODUCTION

Rapid pandemic spread of Covid‐19 has outpaced hospital and healthcare systems’ ability to respond. They have had to adapt quickly to provide testing, treatment and contact tracing for those infected or at‐risk. Concurrently, they have removed vulnerable populations from potential exposure, thus deferring all non‐essential care. Mobilizing healthcare's digital revolution^1^ and rapid conversion to virtual practices^2^ have been deemed essential with virtual care becoming a new default worldwide^3^ both for those who may have Covid‐19^4^ and other patient groups.^5^ Individual telephone consultations have expanded rapidly to triage clinical needs, monitor, coordinate testing and treatment. Although essential, these have limited engagement and require frequent repetition of core messages to each patient. Video consultations improve engagement but do not address repetition or embed peer support.^6^

## 2 VIRTUAL GROUP CONSULTATIONS

Some limitations are addressed by virtual *group* consultations: the overarching term encompassing ways of delivering care virtually with groups of patients rather than one‐to‐one, including virtual/video shared medical appointments.^7^ During this pandemic, innovative global solutions are required. Virtual group consultations can provide better access to services, decrease healthcare rationing, avoid healthcare provider burnout and may result in better health outcomes. Given the rapid and widespread implementation of virtual care during this pandemic, methodologically sound observational studies and clinical trials to test safety and effectiveness should be promoted now.

## 3 PUBLISHED EVIDENCE

The strongest published evidence on virtual group consultations comes from one small (N = 100) non‐randomised mixed‐methods study of a pharmacist plus nurse practitioner‐delivered model for managing patients with diabetes (HbA1c ≥ 7%).^8^ Patients received virtual shared medical appointments or usual care and were followed for 5 months. Virtual groups were small (4‐6 patients), with four weekly sessions, followed by bimonthly refresher sessions. There was significant reduction in HbA1c (–0.8%, *P* = .03), emergency department visits (1 vs 16, *P* < .01) and blood pressure (9/5 mmHg, *P* < .05) in virtual groups. Investigators reported non‐significant trends to reduce hospital admission (1 vs 4), low‐density lipoprotein (LDL) (–0.2 mM) and triglycerides (‐0.5 mM) (all *P* > .50). Patients and healthcare providers were satisfied with virtual shared medical appointments, which were perceived as culturally sensitive and effective in reaching geographically and socially isolated patients (mainly male, veteran, Asian/Pacific Islanders). Peer support (an identified theme) could be optimised, improving efficiency with larger groups (eg up to mean n = 23 primary/secondary care for in‐person groups, based on results from other studies).^7^ This study also demonstrates the flexibility inherent in virtual groups by delivering care in Guam by health professionals 4000‐8000 miles away in Hawaii and Rhode Island.

One other small (N = 66) study compared virtual shared medical appointments with propensity‐matched in‐person attenders with obesity, showing comparable significant weight loss at 3 months (–4.5%, *P* = .97) and 6 months (–4.3% and –5.8%, *P* = –.37).^9^ Models developed in response to the pandemic are starting to be published now showing this approach is generalisable for physical and mental health care.^10^

## 4 UNPUBLISHED EVIDENCE

There is unpublished evidence of virtual group consultations from Cleveland Clinic, where most clinical services have offered offer face‐to‐face shared medical appointments since 2010. More than 160 000 shared appointments have been delivered with patient satisfaction and clinical outcomes as good or better than usual care. Twelve months ago, Cleveland Clinic began virtual appointments for <1% of patients, conducting 120 sessions over 2019. In response to Covid‐19, they scaled rapidly to >27% of patients receiving virtual appointments in the past month, which is translating into rapid expansion of virtual group consultations; prospective data collection is ongoing (Sumego, personal communication). Virtual group consultation can overcome known barriers to care, including socioeconomic inequality, technological limitations, payment models and scepticism.^11^

A holistic approach to health care must incorporate mental and physical health: social distancing must not result in social isolation. The UCSF Department of Psychiatry is now delivering nearly 100% of adult and child outpatient mental health care virtually (Matthew State, personal communication; https://psychiatry.ucsf.edu/news/apps-expert-advice-invaluable-resources-well-being-during-coronavirus-pandemic). UCSF includes virtual one‐to‐one care and virtual groups to assure timely access to critical services especially when physical access is limited during this pandemic. Group care includes pre‐existing treatment groups that have gone fully virtual and novel high‐stress support groups. This provides a model for implementation of virtual groups in mental health as well as other chronic disease specialities.

## 5 WHY NOW?

Cleveland Clinic and UCSF demonstrate feasibility to scale‐up. However, we would not typically extrapolate such limited data to call for virtual group consultations for other conditions and/or populations. Nonetheless, during this global Covid‐19 pandemic, innovation is urgently needed. Hospitals and health systems are crippled by demand, with no capacity beyond emergency care. All but the most urgent outpatient appointments have been cancelled. In many places, primary care has been substituted entirely by telephone consultation – *despite similar lack of evidence*.

## 6 CLINICIAN DEMAND

After receiving numerous enquiries about virtual group consultations in the time of Covid‐19, we came together to share best virtual group consultation practice with other healthcare providers. Our goals were to consult on how best to:

- manage Covid‐19 symptomatic patient triage safely and efficiently;
- provide continuity of care and peer support for chronic disease patients normally seen face‐to‐face;
- consider virtual groups for antenatal care, which cannot be suspended;
- motivate/engage self‐isolating/infected staff to contribute by safely delivering care;
- inform and empower patients at high risk for Covid‐19; and
- assist post‐Covid‐19 pandemic care for patients with deferred routine care.

## 7 WEBINARS

An initiative supported by major international lifestyle medicine organisations to share best practice and support clinicians worldwide to deliver virtual group consultations had strong uptake within the first months of the COVID‐19 pandemic. We organised three 60‐min webinars on 9 April 2020 (https://bslm.org.uk/vgc), inviting foremost group consultation model experts to contribute their virtual experience. Of the original 718 registrants, one‐half (n = 347; 48.3%) had conducted in‐person group consultations; of these, one‐third already had begun experimenting with virtual groups (n = 116 of 347; 33.5%). They planned to use virtual group consultations for a wide variety of purposes including: Lifestyle Medicine/Wellness, Chronic disease management, Various individual acute/chronic disease groups, Diabetes, General practice/general health, Nutrition, Mental health/wellbeing, Educational uses, Weight loss, Antenatal/postnatal/women, All types of care and COVID‐19. Registration by continent was as follows: North America 313 (44%), Europe 192 (27%), Asia/Australasia 185 (26%), South America 19 (3%) and Africa 9 (1%).

Given demand, five additional webinars were conducted between April and November. All content has been shared on the British Society of Lifestyle Medicine website (https://bslm.org.uk/vgc/). A total of 1546 people from more than 50 countries registered for these webinars, with more than 2500 views.

Webinars were rated highly by participants for quality (median = 9/10; interquartile range [IQR] = 8‐10) and likelihood of changing clinical practice (median = 8/10, IQR = 6‐9). Polling during the webinars showed 23% were willing to nominate one or two patient champions, emphasising this model of care is a partnership with patients and co‐design a core element.^12^

## 8 ADVANTAGES

Virtual groups may garner the advantages of face‐to‐face group consultations.^7^, ^13^, ^14^, ^15^ They allow time and space for effective engagement. Patients get more time with their clinicians with opportunities to get questions answered, while embedding peer support. Clinicians of all disciplines can deliver virtual groups after short virtual training (out‐of‐hours or while self‐isolating), leveraging workforce capabilities. Virtual shared medical appointments could enable providers to see two to 10 times more patients, without compromising quality. Healthcare rationing and catch‐up work are avoidable by transitioning to chronic care delivery now. Eliminating repetition of normative advice prevents burn‐out.

This is recognised as quality healthcare provision and billable (eg time‐based models, fixed fee‐for‐service). Signed waivers and adequate technical security must be implemented to address patient confidentiality and privacy concerns. Virtual platforms are familiar to most in developed and even developing nations, though not available to all. We expect increased compliance by eliminating barriers of travel and time needed for routine in‐person care, especially now. Virtual health care has been rapidly expanding to account for physical distancing and reduce risk of infection: transforming health care in the United States, the United Kingdom, Italy, Germany, China, India and South Africa.^3^

## 9 LIFESTYLE AND MORE

The Lifestyle Medicine Global Alliance (https://lifestylemedicineglobal.org/sister-organizations/) supports and promote group consultation models. Therefore, partners in this effort might be drawn from this rapidly growing lifestyle medicine movement and accreditation of healthcare providers in the United States, Europe, Australia and elsewhere, but are also applicable more widely. Evidence‐based areas or ‘pillars’ that can be promoted during virtual group consultations include, but are not limited to, healthy eating, more exercise, managing stress, forming/maintaining good relationships, improving sleep and avoiding smoking and other harmful substances. These public health measures are embedded within most international Covid‐19 guidance. There are no data yet on whether this will change behaviour positively: the immediate focus is rightly on prevention, testing, treatment and risk reduction. Perhaps over time, these healthy behaviours (especially an emphasis on daily exercise) could improve health, analogous to wartime rationing improving nutrition.

## 10 EDUCATION AND ROLE EXTENSION

The pandemic has also had massive impact on both undergraduate and post‐graduate education. This has included service pressures and the need for social distancing both restricting educational opportunities and compelling an increasing proportion of education being delivered online.^16^ Virtual teaching has both advantages and disadvantages,^17^ with advantages including the variety and accessibility. Virtual group consultations are a very feasible teaching platform and are one of the ‘new interactive forms of virtual teaching… to allow patient interaction from the student's home’.^17^ Given that student numbers attending in person group consultations has been a limiting factor for use of this model in undergraduate and interprofessional education,^18^ the ease of groups of students from the same or mixed disciplines attending and potential for small groups discussion in breakout rooms are further advantages.

This is also an opportunity for teams to work more closely and effectively together, rather than into silos, and for receptionists, health care assistants and physicians associates to extend their roles as group facilitators and spend time delivering care with clinicians. So postgraduate education is also supported^7^ and there is now a pathway to accreditation for in person and virtual group consultation facilitators (https://bslm.org.uk/group-consultations-facilitator-accreditation/), enabling them to secure recognition for their knowledge and skills.

## 11 SCALING AND ADOPTION

In addition to Covid‐19 prevention and care, it is critical to maintain systems for those who need care for non‐infectious disease during this epidemic, those who are pregnant or those struggling to manage with on‐going chronic disease or mental illness. Virtual group consultations have the potential to deliver more care to more people, provide peer support which is lacking during this time of social isolation and prevent burn‐out by re‐engaging clinicians from multiple disciplines in collaborative care. This is another example of clinicians leading service reconfiguration during this crisis.^19^ We invite healthcare workers to adopt and patients to ask for virtual group consultations; we believe that they are as good or better than current alternatives. Implementation resources have been developed, for example resources for virtual and in person group consultations across different settings developed with the support of Sir Jules Thorn Trust, and these can be used freely in exchange for key data to build the evidence base (https://bslm.org.uk/vgc). In addition, we must implement integrated data systems to measure efficiency, effectiveness and outcomes compared to telephone consultations, one‐to‐one video consultation and in‐person visits. Funders should recognise and incentivise adoption of virtual group consultations.

## 12 CONCLUSIONS

Interest in group consultations before Covid‐19 was driven by two key editorials 3 years ago.^19^, ^21^ The same four key drivers are required for widespread virtual adoption: (1) system‐specific evidence of value; (2) easy ways to pilot/adapt models; (3) regulatory change/incentives and (4) relevant patient/clinician education. Experience to date from the Cleveland Clinic and UCSF along with adoption now estimated >1000 centres and intent to implement from those attending webinars provides the opportunity to build an evidence base. It is important to evaluate to what extent virtual group consultations confer greater access, continuity of care, peer support, timely information about COVID‐19 and result in better health outcomes. It is intriguing that scientific convergence and this crisis are leading video consultation and group consultation researchers to both move into the virtual group consultation field along with many others. Cross‐fertilisation and collaboration have the potential to boost productivity and implementation science, scaling up to spread essential health innovations.^22^ We must set aside inertia. Given rapid and widespread implementation of virtual care during this pandemic, methodologically sound observational studies and clinical trials to test the safety and effectiveness of virtual group consultations should be promoted now.

## CONTRIBUTORS AND SOURCES

FB had the idea for the article and is guarantor. The manuscript was drafted by FB and JI, with critical input and review by all authors, who agreed the final version and are jointly accountable for its contents. Each author has more than a decade of group consultation experience in their own field (aside from the patient panel lead who has > 1 year) and has important experience of training for delivering or assessing virtual group consultation models. The authorship team is international, multi‐professional and collaborative, just like the virtual group consultation model espoused and the Lifestyle Medicine readership.

## PATIENT INVOLVEMENT

Patient involvement is integral to the co‐design of group consultation models, including virtual group consultations. Our patient panel lead for group consultation research Linda Pepper (a current patient) was invited to attend the webinars and input at the planning stage for this article. She actively participated in the webinars, contributed to the Q and A, and advocated both the importance of wider patient participation and need to clearly define the patient champion role. Linda was also very clear that this article needed to be both open access and accessible to patients, which informed both the choice of journal and analysis article format. Webinar attendees were asked whether they were willing to nominate patient champions for a virtual group consultation patient panel (data below).

## CONFLICTS OF INTEREST

The authors have read and understood Lifestyle Medicine policy on declaration of interests and have the following interests to declare: All authors have completed the ICMJE uniform disclosure form at www.icmje.org/coi_disclosure.pdf and declare no support from any organisation for the submitted work. FB has received research grants for spread and evaluation of group consultations from Sir Jules Thorn Trust, National Institute for Health Research, Medical Research Council and is Editor‐in‐Chief for the Wiley open access journal Lifestyle Medicine. AM is company director of Group Consultations Ltd, an organisation providing paid and unpaid training and implementation support for NHS organisations. AH has received funding for research on group antenatal care from the National Institute of Health Research. LP reports personal fees from MHRA Women's Health Expert Advisory Group, outside the submitted work, is the Patient and Public Involvement editor for BMJ Sexual & Reproductive Health and a public governor for Northumbria Healthcare NHS Foundation Trust. No other relationships or activities that could appear to have influenced the submitted work.
